# Attention Deficit Hyperactivity Disorder in a Male Medium Secure Unit

**DOI:** 10.1192/bjo.2022.379

**Published:** 2022-06-20

**Authors:** Rachel Swain, Zara Azim-Naqvi, Sinthujah Balasubramaniam, Maja Dujic

**Affiliations:** West London NHS Trust, London, United Kingdom

## Abstract

**Aims:**

The worldwide prevalence of Attention Deficit and Hyperactivity Disorder (ADHD) in the adult population is estimated to be 2.5%. Prevalence studies have shown rates to be consistently around ten times higher in the prison population, but there is less known about secure psychiatric hospital populations. ADHD has relevance as a predictor for offending, for challenging behaviours when incarcerated, for lower quality of life and high costs for both the NHS and prison systems. This service evaluation aimed to establish estimated prevalence of ADHD within one male medium secure unit.

**Methods:**

A cross sectional review of computerised medical records for all service users on the male medium secure forensic unit took place, to identify those who met inclusion criteria. Service users who were too acutely unwell or had an established or pending diagnosis of ADHD were excluded.

The Brief Barkley Adult ADHD Rating Scale (B-BAARS), a 5 minute screening questionnaire, was given to service users to complete. Anonymised responses were converted to electronic format and the compiled results analysed.

**Results:**

There were 125 service users at the time of information gathering, with 112 eligible according to inclusion and exclusion criteria. 2 of the excluded service users already had an ADHD diagnosis. 70 service users out of those approached, agreed to take part in the screening (62.5%). 2 out of 70 (2.9%) service users met criteria for a possible diagnosis of ADHD.

**Conclusion:**

Using the B-BAARS, 2.9% of service users on the male medium secure forensic unit reported clinically significant symptoms suggestive of a diagnosis of ADHD. This estimate is significantly lower than other studies in prison settings. When combined with the figure for service users with a pre-existing diagnosis, however, the figure is still higher than in the general population (5.5% compared to 2.5%), and illustrates that screening tools can have a useful function in forensic settings.

There may have been methodological issues with this evaluation, including the self-reported nature of symptoms, the comparatively high level of functioning required to complete the questionnaire and the low response rate amongst the service users.

This evaluation serves to increase awareness about ADHD in the forensic population in general. It also highlights the value of this simple screening tool, or one similar, to clinical teams on the forensic wards. The screening tool could be further utilised in low secure and women's services to establish if results are similar amongst these populations.

